# “Instrução sobre a escolha, preparação e remessa das sementes e cebolas das plantas, que se mandarem vir de África e do Brasil”

**DOI:** 10.1590/S0104-59702023000100009

**Published:** 2023-04-03

**Authors:** Magnus Roberto de Mello Pereira

**Affiliations:** i Professor aposentado do Programa de Pós-graduação em História/Universidade Federal do Paraná. Curitiba – PR – Brasil. magnusdocs@gmail.com

**Keywords:** história da botânica, viagens filosóficas, instruções de remessas botânicas, history of botany, philosofical journeys, instructions on botanical shipment

## Abstract

Manuscrito inédito, datado de 1802, que trata dos métodos a utilizar na recolha e remessa de sementes, tubérculos e bulbos das colônias da África e do Brasil para o Complexo de História Natural da Ajuda, em Portugal.

## Abstract (en)

Unpublished manuscript, dated 1802, dealing with methods to be used in collecting and shipping seeds, tubers and bulbs from colonies in Africa and Brazil to the Natural History Complex of Ajuda, in Portugal.

Entre os papéis referentes a Moçambique, que integram o acervo do Arquivo Histórico Ultramarino, em Lisboa, encontra-se um manuscrito ainda inédito que trata dos métodos a utilizar na recolha e remessa de sementes, tubérculos e bulbos das colônias para Portugal. A instrução, datada de 18 de outubro de 1802, vem assinada por Antônio José de Carvalho Chaves, que era secretário de Governo de Moçambique e chegara à colônia naquele exato ano. Formara-se em cânones pela Universidade de Coimbra em 1799 e não parece ter tido maiores interesses pelas ciências naturais. Sua formação na área devia-se à reforma pombalina da Universidade de Coimbra, de 1772, que obrigava todos os estudantes, independentemente de seus cursos, a frequentar disciplinas de ciências naturais, física e química (Cruz, 2004, p.113; Costa, 2014, p.185-186).

A percepção da carência de administradores preparados para a modernização do Império levou a Coroa portuguesa a adotar um projeto de formação de quadros dotados de conhecimentos científicos. Daí o caráter público e secular da reforma, que objetivava gerar uma universidade “nova”, pensada como parte do aparelho de Estado, à qual foi atribuída a responsabilidade pela formação das novas mentalidades destinadas a colocar Portugal nas sendas do progresso.

Conforme indicado na carta de encaminhamento, a *Instrução* era fruto de uma incumbência dada pelo então ministro da Marinha e Ultramar, o visconde de Anadia. O ministro, por sua vez, transferia a iniciativa ao príncipe regente dom João. É possível supor que, nessa pequena trama palaciana, houvesse o dedo de dom Rodrigo de Souza Coutinho, que deve ter mobilizado o príncipe a solicitar a Anadia a elaboração da instrução. Dom Rodrigo acabara de deixar a pasta Ultramar e havia assumido o Erário Régio, mas deixara ao sucessor um ministério com atribuições reduzidas. Uma portaria, de 24 de novembro de 1801, nomeara dom Rodrigo como inspetor dos “Reais Estabelecimentos” (Jardim Botânico, Museu, Casa do Risco e Laboratório Químico da Ajuda), que continuaram sob sua direção direta (Livro de registo..., 1791-1804, p.51).

## Complexos e redes

Manuais e instruções de recolha de “produtos da natureza”, como o elaborado por Chaves, pertencem a uma conjuntura científica muito específica. Em meados do século XVIII, o naturalista sueco Carlos Lineu desenvolveu as premissas daquilo que se tornaria a base epistemológica das modernas ciências da natureza. O paradigma se apoiava no tripé: (1) adoção do modelo taxonômico por ele desenvolvido, (2) expedições exploratórias e (3) criação de complexos de história natural. Esses complexos eram centrais ao processo e passaram a ser reproduzidos por todo lado. Joseph Banks tornou-se figura central dos Royal Botanic Gardens de Kew e assumiu o controle da política de envio de naturalistas nas expedições inglesas despachadas para todos os continentes. Buffon conduziu um processo semelhante de recolha, a partir do Jardin du Roi. Na Espanha, o Real Gabinete e o Real Jardim Botânico de Madri passariam a centralizar a política imperial de recolha e catalogação de espécimes. Em Portugal, o naturalista paduano Domingos Vandelli seguiria a mesma cartilha, ao implantar, a partir de 1768, o Museu e Jardim Botânico da Ajuda. Por toda a Europa, e mesmo nas colônias, novas instalações foram fundadas, e as antigas foram adaptadas para atender às novas demandas (sob a formação desses complexos, ver Pereira, 2016).

As redes criadas para alimentar tais complexos de história natural não eram compostas apenas por especialistas. Os responsáveis por essas instalações mobilizaram o oficialato civil, militar e religioso submetido às coroas europeias, principalmente o das colônias (Pereira, 2013, p.98-99). Também foi chamada a participação de amadores, que desempenhariam um papel importantíssimo na formação dessas instituições (Drouin, 2011, p.35-47). O problema que se colocava era garantir que a contribuição dos curiosos se adequasse às novas exigências científicas. Assim, foram elaborados diversos manuais com vistas a instruí-los. O controle dessas remessas se acentuou na década de 1780, provocando o aparecimento de muitas compilações temáticas, que, em sua maioria, permaneceram na forma de manuscrito (Spary, 2000, p.82). Foi o caso da *Instrução* de Carvalho Chaves (1802).

### Instructio peregrinatoris

Os manuais dedicados a instruir viajantes naturalistas pertencem a uma longa tradição iniciada no final do século XVII. Eles tiveram ampla circulação por toda a Europa, o que levou a um processo de “intertextualidade” que abrangia toda a república das letras. Cada compêndio era resultado de um processo cumulativo decorrente do diálogo com as obras precedentes. Para tentar conhecer o percurso da *Instrução* de Carvalho Chaves é preciso visitar a totalidade desse universo.

Na Inglaterra, a publicação dessa modalidade de instrução científica foi resultado do impulso dado pela Royal Society para estabelecimento de uma história natural baconiana, que privilegiava a observação e o inventário da natureza. Robert Boyle publicou, em *Philosophical Transactions*, diversos artigos sob o título “General heads for the natural history of a country”, posteriormente reunidos em um pequeno livro publicado em 1692. O naturalista John Woodward (1696) publicou anonimamente suas *Brief instructions* destinadas a orientar viajantes naturalistas.

Ao longo do século XVIII, a produção desses compêndios sofreria grande incremento e diversificação de gêneros (Pereira, Cruz, 2011). Um formato corrente foi o das orientações específicas sobre as formas de recolher, preparar e conservar os produtos da natureza coletados por leigos ou em expedições exploratórias (Collini, Vannoni, 2005a, 2005b). René-Antoine Ferchault de Réaumur foi pioneiro nessa área. Dono de um saber enciclopédico, dedicou-se também à entomologia e à ornitologia. Entre 1737 e 1748, Réaumur publicou diversas obras sobre a conservação de pássaros e insetos. A *Instrução* de Carvalho Chaves se enquadra exatamente nessa vertente especializada.

A tradição dos manuais de viagens científicas iniciada por Boyle e Woodward teve sequência quando Eric Nordblad (1759), um orientando de Lineu na Universidade de Upsala, defendeu e publicou a tese *Instructio peregrinatoris*. Outro de seus discípulos, David Hultman (1753), editou um pequeno manual sobre a organização de gabinetes. A disseminação do paradigma lineano fez com que esses manuais alcançassem grande sucesso.

Na segunda metade do século XVIII, as instruções de cunho geral sobre viagens filosóficas – o que e como olhar, instrumentos necessários etc. – acabaram por ser reunidas em novos compêndios que continham também orientações básicas de recolha e conservação de espécimes. O primeiro com essas características foi publicado pelo naturalista amador Étienne-François Turgot (1758), irmão do futuro ministro-geral das Finanças de Luís XVI. A obra, publicada anonimamente em 1758, intitulava-se *Memóire instructif sur la manière de rassembler, de preparer, de conserver et d´envoyer les diverses curiosités d’histoire naturell.* Trazia anexado o *Avis pour le transport par mer des arbres*, de Duhamel du Monceau (1758), texto que se tornaria o grande clássico nessa matéria.

Na sequência, apareceram diversas instruções especializadas. O *Méthode nécessaire aux marins et aux voyageurs*, de M. Marvye (1763), tratava da recolha e conservação de moluscos. A respeito de transporte de plantas e sementes, ganharam notoriedade os guias de John Ellis (1770, 1773). William Curtis (1771) publicou as *Instructions for collecting and preserving insects*. Sobre taxidermia e preparação de espécimes foram publicados os manuais do abade Manesse (1786) e do inglês Edward Donovan (1794).

Numa perspectiva mais ampla, o médico John Coakley Lettson (1772) fez editar *The naturalist’s and traveller’s companion*, no qual, pela primeira vez, foram reunidos num único manual a qualificação do viajante-filósofo, orientações gerais e os métodos de recolha e conservação. Trazia ainda instruções para um levantamento exaustivo de dados sobre a economia, a história e a organização social das regiões visitadas.

Na década de 1770, a Coroa de Espanha iniciou um processo de recolha e estudo da fauna e da flora na escala planetária de suas colônias. O primeiro manual espanhol de instruções deve-se a Pedro Franco Dávila, naturalista que viveu por duas décadas em Paris, onde reuniu um imenso gabinete de história natural (Pereira, 2013, p.102-103). Carlos III comprou o gabinete e o instalou em Madri com o nome de Real Gabinete de História Natural, inaugurando-o em 1776. Nomeou o próprio Dávila como seu diretor, que, no mesmo ano, fez publicar um livreto destinado aos altos funcionários de todo o Império, com orientações básicas de recolha e conservação de “producciones curiosas de Naturaleza” que deveriam ser enviadas ao gabinete matritense (Dávila, 1776). Em 1779, Casimiro Gómez Ortega publicou um opúsculo de orientações sobre o transporte de plantas por mar e terra. Esse estudioso foi o primeiro diretor do novo Real Jardim Botânico de Madri e principal responsável pelas expedições espanholas que se dirigiram às três Américas e às Filipinas.

Já na França, após as instruções atribuídas a Turgot, houve demora na publicação de novos manuais de caráter geral. De acordo com Lorelai Kury (2001, p.91), o Jardin du Roi, transformado em Museum National d’Histoire Naturelle a partir de 1793, passaria a centralizar a rede francesa de recolha de produtos da natureza, mas só em 1818 seus diretores publicaram um manual de orientação geral para viajantes (Thouin, 1818).

## A regras que o filósofo naturalista nas suas peregrinações deve observar

Em Portugal, o naturalista Domingo Vandelli foi responsável por difundir esses manuais entre seus alunos. Também foi um dos responsáveis pela implementação do projeto de viagens filosóficas às colônias portuguesas, para o qual foram convocados diversos estudantes recém-formados da Universidade de Coimbra, todos eles luso-brasileiros. Na fase preparatória do projeto, ele elaborou um rol de instruções às quais deu o título de *Viagens filosóficas ou Dissertação sobre as importantes regras que o filósofo naturalista nas suas peregrinações deve principalmente observar* (Vandelli, 1779). Como tantos outros manuais, o de Vandelli jamais foi publicado. Ele não deixa, no entanto, de ser um dos mais interessantes elaborados no período. Tal como *Traveller’s companion*, de Lettson (1772), ele buscou cobrir diversos aspectos das viagens filosóficas, da importância e de como fazer diários, o que observar e os meios de preservar os produtos da natureza recolhidos.

A edição de um manual destinado a estimular e homogeneizar a recolha em todo o Império ocorreu em 1781, quando a Academia das Ciências de Lisboa publicou as *Breves instruções aos correspondentes da Academia das Ciências de Lisboa sobre as remessas dos produtos e notícias pertencentes a história da natureza para formar um museu nacional* (Breves instruções..., 1781) (sobre os manuais portugueses, ver Brigola, 2003; Pereira, Cruz, 2009; Pataca, 2011). Essa obra acabou adotada pela Coroa portuguesa e distribuída a praticamente todos os governadores e altos funcionários régios dos territórios ultramarinos. Foi o principal texto de orientação da rede mobilizada para a formação do complexo de História Natural da Ajuda (Domingues, 2001). No mesmo ano, os discípulos de Vandelli concentrados na Ajuda elaboraram um manual, sob a coordenação de Alexandre Rodrigues Ferreira (Ferreira et al., 1781), intitulado *Método de recolher, preparar, remeter e conservar os produtos naturais*. Essas instruções ficaram inéditas, no entanto.

A sequência de elaboração de manuais portugueses destinados a orientar viagens filosóficas não cessaria aí. Em 1783, José Antonio de Sá publicou o *Compêndio de observações que formam o plano da viagem política e filosófica que se deve fazer dentro da pátria*. O título indica a preocupação de que as empreitadas filosóficas não visassem exclusivamente às colônias, mas que fossem planejadas expedições em território metropolitano. Sá dividiu o seu manual em duas seções, uma destinada à viagem filosófica, apoiada nas *Instruções* da Academia de Ciências, e outra à viagem política, que hoje diríamos ser de cunho mais institucional, econômico e antropológico. O *Compêndio *foi um dos mais extensos e completos manuais de orientações ao viajante naturalista até então publicados na Europa. É comparável, e mais completo em alguns aspectos, ao *Companion*, de John Lettson.

Em data posterior às *Breves instruções* da Academia, Vandelli incumbiu Agostinho José Martins Vidigal, estudante de medicina em Coimbra, de fazer uma compilação de diversas memórias instrutivas “ilustradas com os melhores métodos de haver, conservar e examinar os diversos objetos da História Natural, e com orientações sobre os meios de recolher utilidade das viagens, principalmente no que respeita às Ciências da Natureza” (Vidigal, s.d.). Na composição de suas instruções, o estudante reporta-se a uma memória sobre recolher produtos da natureza, de “autor desconhecido”, ou seja, a obra de Turgot, publicada anonimamente. Outra instrução referenciada é o *Méthode *de Marvye (1763), sobre a coleta de moluscos. Duhamel du Monceau era a principal referência sobre o transporte por mar de árvores e plantas e sobre preservação de sementes. Vidigal menciona ainda os trabalhos de Réaumur sobre a conservação de “produtos da natureza”. Também alude a dois trabalhos dos “discípulos de Lineu”: David Hultman (1753) e Henrique Nordblad (1759). Indica ainda um manual intitulado *O viajante naturalista*, a versão em francês (*Le voyageur naturaliste*) da obra de Lettsom (1775). Por último, são mencionadas as orientações sobre o transporte de plantas por mar, de Gómez Ortega.

Um dos aspectos de maior interesse das instruções de Vidigal é o fato de ser assumidamente uma compilação. Em decorrência, são explícitas as referências aos textos que serviram de base para a sua elaboração. A bibliografia utilizada dá uma mostra da produção internacional das orientações para viajantes naturalistas e quais estavam sendo lidas em Portugal. Conforme se observa, os estudantes de Coimbra, como Carvalho Chaves, autor da *Instrução*, tinham amplo acesso à literatura mundial especializada sobre o tema. Além disso, já contavam com um corpo de manuais elaborados no próprio país.

O passo seguinte na publicação oficial de instruções em Portugal inscreve-se na tradição das obras sobre procedimentos especializados. Em 1798, a tipografia de Thadeo Ferreira publicou uma *Instrução completa sobre o método de apanhar, manejar, conservar e empacotar os insetos* (Instrução completa..., 1798), muito provavelmente uma tradução apócrifa de obra inglesa ou francesa. Não é possível ter certeza se se tratava de uma iniciativa privada ou oficial, uma vez que essa tipografia era usada por dom Rodrigo de Souza Coutinho para imprimir uma série de obras traduzidas por sua orientação.

Nesse sentido, uma coleção de monografias específicas chegou a ser planejada para publicação pela Casa Literária do Arco do Cego, sob a supervisão de Mariano da Conceição Veloso, intitulada *Naturalista instruído*. Todavia, num primeiro momento, foi publicado apenas um tomo, sobre o reino animal (Veloso, 1800), a tradução do *Traité sûr la manière d’empailler*, do abade Manesse (1786).

A encomenda da *Instrução *de Chaves, de 1802, enquadra-se nessa conjuntura em que se buscava suprir a falta de tutoriais especializados em língua portuguesa. Todavia, até onde foi possível alcançar, suas prescrições não são cópia nem tradução de outras, o que é surpreendente. Elas não reproduzem e não parecem dialogar com as mais conhecidas orientações referentes a transportes de sementes e bulbos, que eram as elaboradas por John Ellis e Duhamel du Monceau. Também não houve apropriações das instruções portuguesas, que, por sinal, avançavam pouco no assunto. Vandelli, por exemplo, não fora além de recomendar condensadamente a tomar precauções contra a umidade e sugeria a técnica proposta por Ellis de encapsular sementes em cera de abelha. Carvalho Chaves sequer se refere a esse método.

Publicação oficial portuguesa de obra sobre a recolha e transporte de vegetais só viria a ocorrer em 1805, com o título *Instruções para o transporte por mar de árvores, plantas vivas, sementes* (Veloso, 1805). A obra era novamente uma tradução, a do *Avis pour le transport par mer des arbres*, de Duhamel du Monceau, do qual foi suprimida a parte final.

Ainda no período colonial, um último manual oficial sobre o tema acabaria por ser editado no Rio de Janeiro, no quadro da transferência da corte para o Brasil. Tratava-se das *Instruções para os viajantes e empregados nas colônias sobre a maneira de colher, conservar e remeter os objetos de história natural* (Thouin et al., 1819), tradução do manual francês já mencionado, publicado em Paris no ano anterior. Como nessa época o Jardim Botânico do Rio de Janeiro estava em processo de implantação, a Coroa achou por bem mandar traduzir as orientações adotadas por seu congênere francês. Impressiona a rapidez, pois apenas um ano separa a edição francesa da tradução publicada no Brasil, na qual foi incluído um longo balanço do estado das ciências naturais em Portugal e suas colônias no exato momento em que as tentativas autônomas de exploração científicas estavam sendo abandonadas em prol da abertura do território para viagens de naturalistas vindos das principais potências europeias.

## Sobre Antonio José de Carvalho Chaves

A biografia do autor da *Instrução sobre a escolha, preparação e remessa das sementes e cebolas das plantas *ainda está longe de ser minimamente estabelecida*.* Sobre suas origens sabe-se apenas que Carvalho Chaves era filho do doutor José Manoel Chaves e de dona Rosa Maria de Carvalho e nasceu em Condeixa-a-Nova, Portugal. Formou-se em cânones (direito canônico) pela Universidade de Coimbra.

Como vimos, a primeira comissão que recebeu da Coroa foi na África, em 1802. Posteriormente, foi nomeado para uma magistratura no Brasil, onde fez uma carreira bem-sucedida e viveu o resto de sua vida. Laurenio Lago (1940, p.47) reuniu alguns dados sobre sua trajetória institucional:

Formou-se em Leis pela Universidade de Coimbra, conforme carta de Bacharel datada de 23 de novembro de 1809.

Em decreto de 13 de maio de 1811, foi nomeado Juiz de Fora da comarca de Cuiabá, obtendo por alvará de 4 de fevereiro de 1812, o lugar de Provedor da Fazenda dos Defuntos e Ausentes, Resíduos e Capelas enquanto exercesse aquele lugar.

Havendo bem desempenhado o mesmo lugar, foi a ele reconduzido com o predicamento do primeiro banco, em decreto de 13 de março de 1815.

Foi nomeado Desembargador da Relação da Bahia, pela imediata resolução de 6 de agosto de 1821, tomada sobre consulta da Mesa do Desembargo do Paço.

Passou para a Casa da Suplicação como Desembargador Ordinário e de Agravos, em decretos de 12 de outubro de 1827 e 18 de outubro de 1829.

Em decreto desta última data, foi nomeado Corregedor do Crime da Corte e Casa.

Com a extinção da Casa da Suplicação ficou pertencendo à Relação do Rio de Janeiro, conforme foi declarado em portaria de 11 de março de 1833 do Ministro da Justiça.

Foi nomeado Ministro do Supremo Tribunal de Justiça, em decreto de 15 de setembro de 1842, na vaga proveniente do falecimento de Euzébio de Queiroz Coutinho da Silva, tomando posse em 27 do mencionado mês.

Foi agraciado por D. Pedro I com o grau de Cavaleiro da Ordem do Cruzeiro, em decreto de 2 de agosto de 1826, o foro de Fidalgo Cavaleiro, em decreto de 18 de janeiro de 1830, e Oficialato da Ordem da Rosa, em decreto de 17 de outubro do mesmo ano, e por D. Pedro II com o título do Conselho, em carta de 26 de setembro de 1842.

O Conselheiro Antonio José de Carvalho Chaves faleceu em Niterói, província do Rio de Janeiro, no dia 29 de julho de 1847, conforme se verifica do registro de óbitos da Igreja de S. João Batista da mesma cidade, sendo sepultado nas catacumbas da Igreja da Conceição.

Esses dados estão basicamente corretos, mas é preciso corrigir alguns pormenores. Lago afirma que Chaves formou-se em leis pela Universidade de Coimbra, em 1809. Na verdade, ele cursou cânones entre 1794 e 1799.^1^

Em 1802, apenas dois anos após se formar, recebeu o Hábito da Ordem de Cristo (Diligência de habilitação..., 13 mar. 1802) e ganhou o cargo de secretário de Governo em Moçambique, o que é um indicativo de que era “bem nascido”. Foi para aquela colônia na condição de letrado, e não com uma patente militar, como costumam afirmar. Quando voltou a Portugal, foi agraciado com a magistratura de juiz de fora de Cuiabá. Sua chegada ao Mato Grosso ficou anotada nos *Anais do Senado da Câmara de Cuiabá* (Anais..., 2007, p.202).

No dia 30 de Outubro [de 1812] chegou a esta vila com feliz sucesso o Dr. Juiz de Fora Antônio José de Carvalho Chaves, cavaleiro professo na Ordem de Cristo, e não entrou no governo da justiça por ser haver dado parte da sua chegada, como é de costume, ao Ilmo. e Exmo. Sr. Governador e Capitão-general. … No dia 10 deste mês deu posse o Senado da Câmara ao Dr. Antônio José de Carvalho Chaves do cargo de Juiz de Fora em consequência da régia provisão que apresentou, na presença da nobreza e povo que assistiram a este solene ato, e igualmente foi empossado do cargo de Provedor das Fazendas dos Defuntos e Ausentes, Capelas e Resíduos, em consequência de outra régia provisão que apresentou; e neste mesmo ato levantando-se da sua cadeira o dito Dr. Juiz de Fora, fez uma elegante fala.

Chaves manteve-se naquela capitania por uma década. O padrão de permanência desses oficiais em colônias longínquas e desprestigiadas, como Mato Grosso, era de três anos (Almeida, 2018, p.289). Por meio de sucessivos requerimentos, ele tentou abreviar sua permanência no sertão mato-grossense. Solicitou, sem sucesso, a transferência para Pernambuco, Sabará, Mariana, Itú e Taubaté (Chaves, 1815-1829). Mais felizes foram seus pedidos de promoção local. Foi reconduzido ao cargo de juiz de fora de Cuiabá e, em 1818, promovido a ouvidor da capitania (Relação dos despachos..., 13 jan. 1819, p.3).

Durante sua longa estada em Mato Grosso, constituiu um núcleo familiar. Aparentemente, não casou, pois a documentação jamais se refere a uma possível esposa. Todavia, sabe-se que teve diversos filhos. Chaves (2018) deixou um manuscrito, escrito originalmente em 1819, parcialmente inédito, denominado *Curiosidades em Cuiabá*, em cujas páginas sobrantes um de seus filhos anotou diversos dados sobre a família. Lê-se no manuscrito que ele “Perfilhou filhos legítimos a Antônio Lucas Chaves, Luiz Antônio Chaves e Antônio [?] Maria Chaves todos filhos da cidade de Cuiabá na Província de Mato Grosso” (citado em Mourão, 2008, p.73). Desse trecho, pode-se deduzir que ele legitimou alguns filhos tidos em Cuiabá fora do casamento.

O final da permanência de Chaves em Mato Grosso coincide com a complicada conjuntura da Revolução Liberal e a instalação das Cortes em Portugal. Conjunção que ali foi duplamente complexa, pois ocorreu uma crise institucional entre Cuiabá, a capital de fato, e Vila Bela, que legalmente era a cabeça da capitania. Com a expulsão do capitão-general, foi eleita uma junta governativa em Cuiabá. Após a morte do primeiro presidente da junta, foi feita nova eleição, em agosto de 1822, na qual Chaves foi eleito presidente (D’Alincourt, 1880-1881, p.95-96). Permaneceu no cargo até julho de 1823. A atuação nesse período no governo da capitania foi por ele bem inflada e serviu para alavancar suas pretensões futuras. Ao solicitar a Ordem do Cruzeiro, argumentaria que no governo provisório de Mato Grosso “advogou a causa da independência e do Império” (Chaves, 1815-1829), levando as vilas de Cuiabá e Mato Grosso a aderir à causa da Independência (Silva, 2014, p.308-309). Por seus supostos feitos, foi condecorado com a Ordem do Cruzeiro, em 12 de outubro de 1827, por ocasião do aniversário do imperador Pedro II (Relação dos despachos..., 16 out. 1827). Chaves foi desembargador dos agravos e corregedor do crime da Corte, até 1842, quando foi nomeado ministro do Supremo Tribunal de Justiça e passou a integrar o Conselho de Estado, recém-restaurado por Pedro II. Faleceu em Niterói, em 1847, no exercício do cargo.

**Figura 1:**
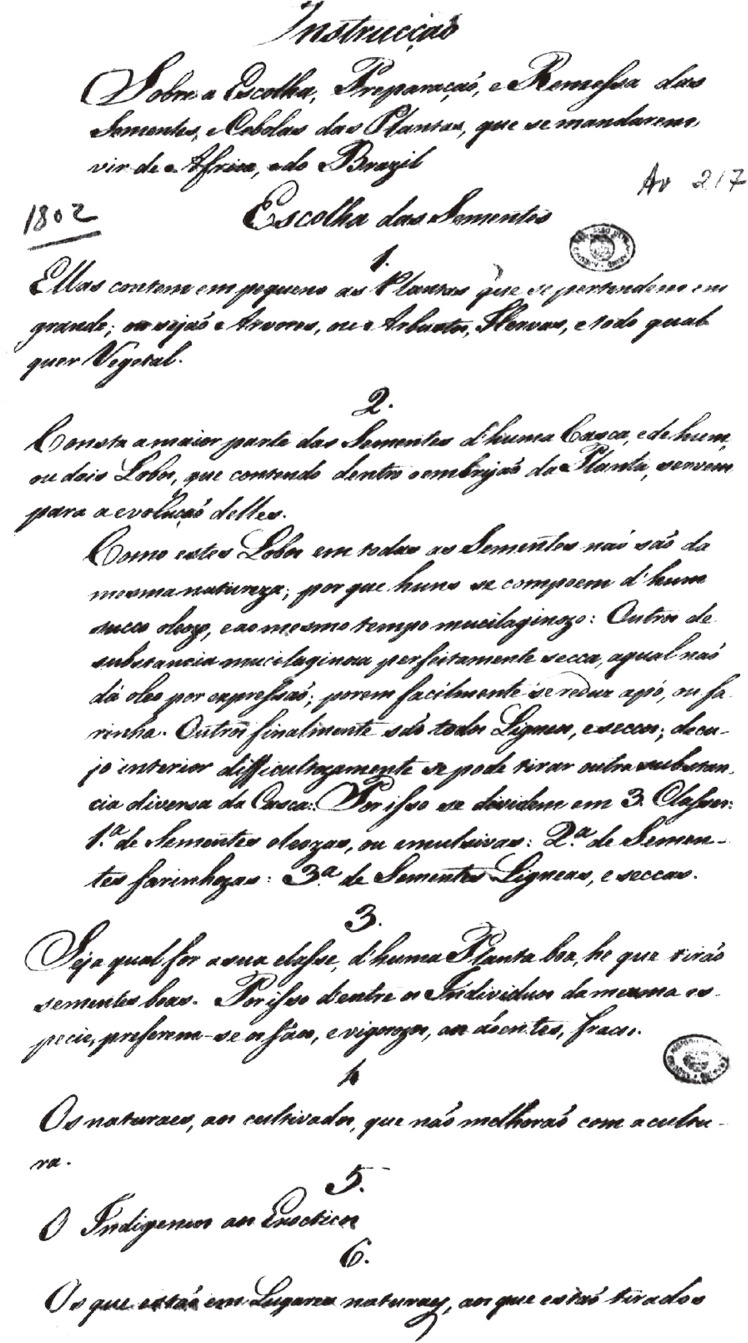
Instrução sobre a escolha, preparação e remessa das sementes e cebolas das plantas, que se mandarem vir de África e do Brasil (Chaves, 1802)

## Footnotes

1 Consulta sobre a inscrição em disciplinas e formaturas dos estudantes coimbrãos pode ser feita no Índice de alunos da Universidade de Coimbra (Índice de alunos..., s.d.).

## Documento

### Documento

[p.1]

Sua [danificado] Regente Nosso Senhor [danificado]

que V. S.ª encarregando algumas Pessoas, inteligentes de Botânica, de indagar quais são as Plantas mais raras, que vegetam nessa Capitania; remeta a este Reino as melhores Sementes e Cebolas que se encontrem das mesmas Plantas, observando para sua condução o método indicado na inclusa Nota, afim de que venham bem acondicionadas e conservem sempre a sua força.

Deus Guarde a V S.ª Palácio de Queluz em 11 de outubro de 1802 = Visconde de Anadia = Senhor Izidro de Almeida Souza e Sá.

Antônio José de Carvalho Chaves

[p.2]

### Instrução

#### Sobre a Escolha, Preparação e Remessa das Sementes e Cebolas das Plantas, que se mandarem vir de África e do Brasil

##### Escolha das Sementes

1.

Elas contêm em pequeno as Plantas que se pretendem em grande; ou sejam as Árvores, ou Arbustos, Ervas e todo qualquer Vegetal.

2.

Consta a maior parte das Sementes de uma Casca, e de um, ou dois Lobos, que contendo dentro o embrião da Planta, servem para a evolução deles.

Como estes Lobos em todas as Sementes não são da mesma natureza; porque um se compõem de um suco oleoso e ao mesmo tempo mucilaginoso: Outros de substância mucilaginosa perfeitamente seca, a qual não dá óleo por expressão; porém facilmente se reduz a pó, ou farinha. Outros finalmente são todos Lígneos e secos; de cujo interior dificultosamente se pode tirar outra substância diversa da Casca. Por isso se dividem em 3. Classes: 1ª. de Sementes oleosas, ou emulsivas: 2ª. de Sementes farinhosas: 3ª. de Sementes Lígneas e secas.

3.

Seja qual for a sua classe, de uma Planta boa é que virão sementes boas. Por isso dentre os Indivíduos da mesma espécie, preferem-se os sãos e vigorosos, aos doentes, fracos.

4.

Os naturais, aos cultivados, que não melhoram com a cultura.

5.

Indígenas aos Exóticos.

6.

Os que estão em Lugares naturais, aos que estão tirados

7.

Cujos troncos, sendo árvores, são os mais grossos e mais bem conformados; de alta e bela raça; sem vício, ou doença alguma procedida, ou de erro de constituição interior, ou de acidentes externos; como são geadas fortes e queimadas; furacões e ventos impetuosos; incisões e cortes violentos; Insetos e Plantas parasitas.

8.

Que os seus frutos sejam bem nutridos e maduros; com o sabor e cheiro e a cor naturais e não alterados; a carne sã sem ser bichosa, nem fredrenta [*sic*]: Que eles são os requisitos para darem boas sementes.

9.

Elas se colherão em tempo seco e não úmido.

10.

Nem verdes, nem inchados, nem engelhados; ultimamente nem velhos, nem podres.

11.

Só as bem nutridas e maduras; escolhendo as grandes e cheias; inteiras e não partidas, ou quebradas.

12.

Que não estejam mordidas dos Ratos e das Formigas; nem furadas do Gorgulho, nem picadas das Vespas e outros Insetos.

13.

Que sacudidas elas não larguem pó.

14.

Que sendo maciças e pesadas vão ao fundo dentro d’água e não nadem em cima dela.

##### Preparação e Exsicação

15.

O seu fim é privar as Sementes de humidade supérflua para as conservar por mais tempo, os meios são os Seguintes.

16.

Sendo Grãos, como Trigo e Cevada, escolhem-se os das primeiras espigas e primeiro se secam, de que se debulhem. Joeira-se quando são grandes quantidades para se alimparem do pó e da moinha das Cascas e passam-se pelo Crivo para se [ilegível]

[p.3]

[danificado] e mais bem vingado. [danificado]

embrulham-se em papel forte e guardam-se em lugar [danificado] se remeterem.

17.

Sendo Legumes, como Favas, Feijões, ou se colhem com as mesmas vagens, quando chocalham dentro delas, ou abrindo-se elas se apanham os que caem por si mesmos. Aos polposos, como os Tamarindos, tira-se-lhes toda a substância mole onde se envolvem as Sementes, mas sem passar pela decocção.

18.

Sendo Pevides, como de Melão, Melancia e outros frutos carnosos e suculentos; separam-se igualmente da sua carne, estando ela bem madura, para não apodrecerem juntamente.

19.

Sendo Caroços, como das Ameixas e Damascos, não se lhes quebra a casca grossa de fora para se lhes tirar a amêndoa se dentro, nem esta se despe da sua pele.

20.

Para se conservarem estas e outras Sementes, espera-se por algum tempo ou ao calor do Sol, ou sobre Fornos e Estufas até secá-las e não torrá-las. Secar e tirar-lhes a humidade supérflua e a necessária a fim de destruí-las. Este mal não lhe faz calor do Sol, nem das Estufas, sendo ele moderado, como a precisão; particularmente as Sementes oleosas, ou emulsivas para se não fazerem rançosas. As farinhosas e secas, com bem pouco calor se secam.

A maior parte das Antigos e ainda mesmo dos Modernos, recomenda que as folhas e as Sementes das plantas, não se sequem senão à sombra e lentamente. Mas a observação não autoriza este método. Observa-se que os que têm poucos princípios resinosos secando-se lentamente fazem-se negras e perdem muito das suas virtudes. As que abundam daqueles princípios não perdem tanto como as aquosas; mas sempre muito mais do que as que se secassem rapidamente. Com facilidade o comum das Plantas atrai a umidade do ar e criam bolor. O que não acontece ás que se secam rapidamente ao calor; que até ficam conservando as suas cores vivas, e brilhantes; o cheiro e outras propriedades essenciais. Ao Sol é que por

[p.4]

[danificado] e das Árvores silvestres [danificado] colher [danificado] secas à sombra; e elas por si mesmas caem e se reproduzem.

##### Cebolas

21.

O tempo de as colher é quando perdem a rama.

22.

O modo é cavando a terra em roda com um Sacho sem ofender a Cebola, para a tirar com torrão.

23.

Sacode-se-lhe a terra fora; tiram-se-lhe as folhas e cascos velhos; e cortam-se-lhes as barbas, ou raízes filamentosas.

24.

As melhores são as maiores, não estando elas greladas, ou tocadas.

25.

Alastram-se em algum tendal, ficando cada uma sobre si, sem estarem amontoadas, ou muito juntas umas das outras.

26.

Expõem-se ao calor do Sol, ou as Estufas, movendo-as várias vezes por dia, para se renovarem as Superfícies.

27.

Ainda mais facilmente se secam as Batatas.

##### Remessas

28.

Sendo Sementes e fáceis de se embrulhar; cada uma das suas espécies se embrulha à parte em seu papel grosso e encerado, podendo ser, vindo cada espécie diversa necessariamente separada da outra, de sorte que se não confundam.

29.

Nota-se em algum bilhete junto, ou o número correspondente ao da Relação que acompanhar a remessa, ou precisamente o mesmo que se precisa saber a respeito de cada Planta. Isto é.

30.

O seu nome sistemático e trivial, se o tiver.

31.

A sua proporção: se é Arvore, ou Arbusto, Erva, e outro qualquer Vegetal

[p.5]

[32. Danificado]

A sua duração: Se é de um, ou dois anos [danificado]

33.

O Lugar onde nasce: Se é aquático, ou terrestre; campestre, ou musgoso; seco, ou pantanoso.

34.

A terra onde se cria: Se é preta areenta, ou barrenta; fina ou forte.

35.

Quando floresce ou frutifica.

36.

Ultimamente o seu préstimo: Na Economia Rural, na Medicina

37.

Com estas declarações acima, uns poucos de embrulhos de sementes atados entre si fazem um, ou mais maços, que se empacotam em papel forte de folha dobrada e encerada. Uns poucos de maços deste se acomodam em uma, ou mais Bocetas, não havendo Caixas de Folhas de Flandres, que preservam melhor da humidade.

38.

Uma grande parte das Sementes amadurece e se seca dentro das suas cápsulas; onde mais facilmente se conservam; e, portanto, mais conveniente é, e mais cômodo mandar as mesmas cápsulas com as suas sementes e acomodando-as como se disse Nº 37.

39.

Outros remetem as Sementes dentro em garrafas e outros vasos de vidro bem tampados e lacrados: cujo método é seguro.

40.

Também se remetem as sementes grandes entre areia do Rio fina e bem seca; e deste modo é que se mandam as Cebolas.

41.

As quais estando greladas e não havendo outras para se remeterem dispõem-se em Caixões de terra, que se cava no lugar

[p.6]

[danificado – linha inteira]

chegam ao lugar do seu destino

42.

Por conclusão, as mesmas Sementes e raízes bulbosas se remetem acamadas entre feto, ou musgo fresco e não úmido, nem calcado.

// 18 de outubro de 1802 //

Antônio José de Carvalho Chaves
